# The Association of Neuroendocrine Differentiation with MicroRNA 21 and MicroRNA let7f Expression and the Clinicopathological Parameters in Primary Invasive Breast Carcinomas with Neuroendocrine Features

**DOI:** 10.3390/diagnostics14192211

**Published:** 2024-10-03

**Authors:** Gamze Usul, Esra Canan Kelten Talu, İsmail Yılmaz, Gizem Narlı Issın, Sibel Bektaş, Didem Can Trabulus

**Affiliations:** 1 Department of Pathology, Istanbul Training and Research Hospital, Hamidiye Faculty of Medicine, University of Health Sciences, Istanbul 34098, Türkiye; 2 Department of Molecular Pathology, Faculty of Medicine, Institute of Health Sciences, Dokuz Eylul University, İzmir 35410, Türkiye; 3 Department of Pathology, Sultan Abdülhamid Han Training and Research Hospital, Hamidiye Faculty of Medicine, University of Health Sciences, Istanbul 34668, Türkiye; 4 Mengücek Gazi Education and Research Hospital, Faculty of Medicine, Binali Yıldırım University, Erzincan 24180, Türkiye; 5 Department of Pathology, Gaziosmanpaşa Training and Research Hospital, Hamidiye Faculty of Medicine, University of Health Sciences, Istanbul 34255, Türkiye; 6 Department of General Surgery, Istanbul Training and Research Hospital, Hamidiye Faculty of Medicine, University of Health Sciences, Istanbul 34098, Türkiye

**Keywords:** breast carcinoma with neuroendocrine features, miRNA, miR-21, miR-let7f, biomarker, paraffin tissue blocks

## Abstract

MiRNAs have been reported as biomarkers with diagnostic, prognostic, and predictive value for many different diseases. Therapeutic agents targeting some miRNAs are currently being developed. We aimed to compare BC-NEFs (carcinoma of the breast with neuroendocrine features) with IDC (invasive ductal carcinoma) cases without neuroendocrine features in terms of the level of miRNA expression known to show the oncogenic (miR-21) and tumor-suppressor effects (miR-let7f) and the clinicopathological features. A total of 29 patients with a diagnosis of BC-NEFs (15 cases with neuroendocrine differentiation >50% of the whole section of tumor and 14 cases with neuroendocrine differentiation 10–50% of the tumor) and 30 patients with a diagnosis of IDC (no neuroendocrine differentiation) were retrospectively re-evaluated. Expression levels of miR-21 and miR-let7f were determined by the qRT-PCR method in paraffin tissue blocks. MiR-21 expression was significantly higher in the IDC group than in the group with BC-NEFs. miR-let7f expression was significantly lower in the group with BC-NEFs compared to the IDC group. A high expression level of miR-21 was found to be associated with progesterone receptor (PR) negativity. Our findings show that the presence of NEFs in breast carcinomas makes a significant difference in the expression levels of the investigated oncogenic (miR-21) and tumor-suppressor (miR-let7f) miRNAs. These findings suggest that miRNAs may be a potential biomarker in BC-NEFs and would benefit from targeted therapy.

## 1. Introduction

Primary invasive breast carcinoma with neuroendocrine features is a rare histological subtype of breast carcinoma. These tumors were grouped under the main heading of neuroendocrine neoplasia, similar to neuroendocrine tumors of the gastrointestinal tract and lung, and were divided into two subheadings as ‘neuroendocrine tumors’ and ‘neuroendocrine carcinomas’ in the last WHO classification (5th edition) published in 2019 [1]. ‘By definition, they are defined with the presence of diffuse, uniform neuroendocrine marker staining detected in more than 90% of the tumor in a tumor with neuroendocrine morphology’ [1]. These tumors, which were thought to be rare in the past due to the uncertainty in the diagnostic criteria and the lack of use of neuroendocrine markers in routine examination, now have been reported to make up approximately 2 to 10% of all breast carcinomas [2,3,4,5,6,7,8,9]. Similarly, controversial data also existed on their prognosis [10]. BC-NEFs was reported in association with worse clinical outcomes compared to IDC in recent studies [8,9].

The treatment decision in breast cancer patients is currently determined by the molecular subtype characteristics of the tumor, including the hormone receptor expression status (estrogen and progesterone), as well as the HER2 status and Ki-67 proliferation index levels. BC-NEFs are heterogeneous in clinicopathological parameters. Therefore, there is no specific therapy targeting NE differentiation, and all breast carcinomas with any NE differentiation are treated similarly to other invasive breast carcinomas. Thus, the current treatment approach for BC-NEFs comprises surgery in combination with chemotherapy, endocrine treatment, and radiotherapy [11]. Most BC-NEFs show hormone receptor positivity (luminal phenotype) [12,13]. In this regard, a correct definition of BC-NEFs may contribute to the selection of a patient group within the group of patients who will receive hormone therapy, whocould benefit from targeted therapy agents that may have an effect on NEFs.To our knowledge, there areno data from prospective clinical trials for the optimal treatment management of BC-NEFs. However, several clinical studies evaluating the therapeutic potential of anti-miRNAs in human diseases are currently ongoing [14].

miRNAs are single-stranded, non-coding, endogenous small RNAs approximately 18–24 nucleotides long that bind to target messenger RNA (mRNA) by basepairing and suppress gene expression post-transcriptionally (epigenetic silencing) [15,16,17]. miRNAs play a role in many physiological processes such as the cell cycle, cell differentiation and regulation, and apoptosis as well as neoplastic processes. They play a role in the neoplastic process by presenting oncogenic or tumor-suppressor features according to the features of the molecular pathways of the mRNA they target. miRNA levels differ between normal and tumor tissues in different types of cancer [18]. In this regard, miRNAs have been defined as biomarkers in the diagnosis, prognosis, and post-treatment follow-up of diseases. In a related manner, predicting the pathological tumor response after treatment, providing information about possible drug resistance, and developing therapeutic agents that directly target disease-associated miRNAs have become important issues in recent years [19,20,21,22].

We compared BC-NEFs with IDC cases without neuroendocrine features, matched for molecular subtype and stage, in terms of miRNA expression levels that are known to show oncogenic (miR-21) and tumor-suppressor effects (miR-let7f) in addition to the clinicopathological characteristics in this study.

## 2. Materials and Methods

### 2.1. Case Selection

**Figure 1:**
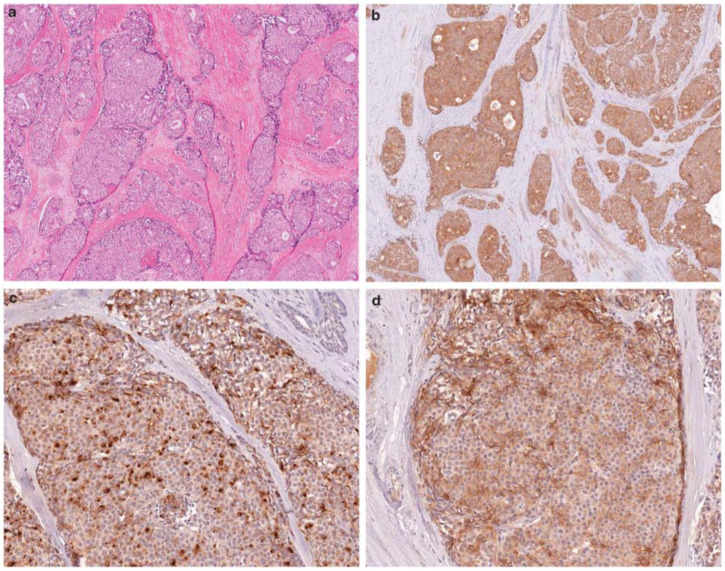
Microscopic images of BC-NEFs. Tumor proliferation is seen as solid nests.(**a**): H&E section of BC-NEFs (×40); (**b**,**d**): diffuse positivity for synaptophysin (×40 and ×100); (**c**): chromogranin positivity (×100).

**Figure 2:**
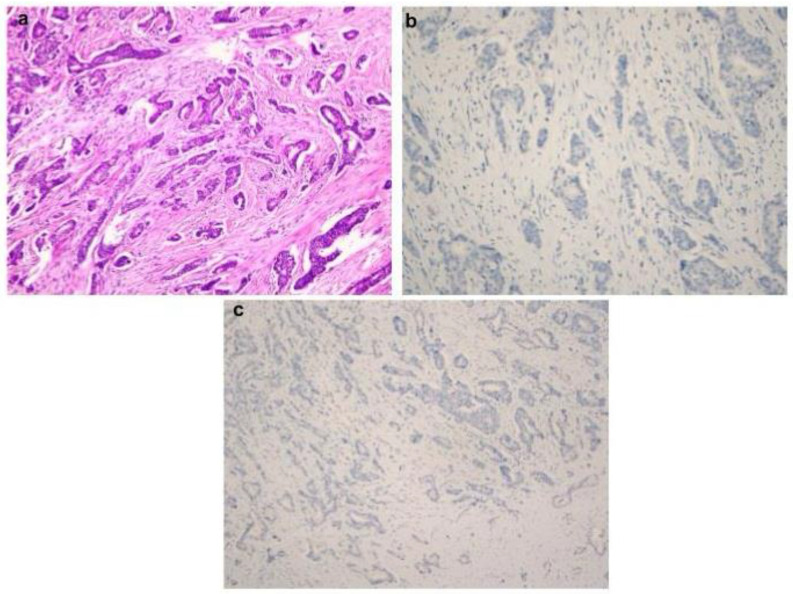
Microscopic appearance of IDC-NOS. Tumor proliferation shows glandular formations. (**a**): H&E section of IDC-NOS (×100); (**b**,**c**): no staining for synaptophysin or chromogranin (×100).

Pathology reports of breast resection materials evaluated at the Istanbul Training and Research Hospital’s Pathology Department between 2012 and 2017 were accessed by reviewing the electronic archives of the hospital. Among these cases, 29 cases diagnosed with BC-NEFs and 30 consecutive cases diagnosed with IDC that matched these cases in terms of molecular subtype and stage but did not have NEFs were identified. Slides and paraffin blocks of all the cases were removed from the archive for retrospective re-examination. We then separated the groups depends on the neuroendocrine differentiation (both tumor morphology on H&E sections and immunohistochemical positivity for NE markers such as synaptophysin and chromogranin) in the whole-cut section of the tumor. Thus, Group 1 was defined as breast carcinoma with NE differentiation in >50% of the tumor (*n* = 15 cases). Group 2 was defined as breast carcinoma with NE differentiation in 10–50% of the tumor (*n* = 14 cases). Group 3 was defined as breast carcinoma with no NE differentiation morphologically and immunohistochemically (negative for both synaptophysin and chromogranin) (*n* = 30 cases). Microscopic images including H&E sections and immunohistochemical stainingsareshown in Figure 1 and Figure 2.

Neuroendocrine tumors metastasizing to the breast, cases with carcinoma in situ only, and cases with a history of neoadjuvant treatment were excluded from the study. No small-cell or large-cell neuroendocrine carcinoma morphology was found among the included cases. All study procedures were performed according to the Declaration of Helsinki principles.

The clinicopathological characteristics of the patients are summarized in Table 1. The AJCC staging system was used for the clinical staging of breast cancers. According to this staging system, breast carcinomas were classified clinically as early breast cancer (clinical stage I, stage II, and some stage IIIA tumors), locally advanced breast cancer (clinical stage IIIB, stage IIIC, and some stage IIIA tumors), and metastatic or recurrent breast cancer (stage IV). Clinical follow-up information of the patients between 2012 and 2024 was noted. It was 71 months (median) for Group 1 (min–max: 7–133), 104 months (median) for Group 2 (min–max: 25–135), and 133 months for Group 3 (min–max: 13–152). Disease-free survival was defined as the time from surgery to recurrences. Overall survival was defined as the time from diagnosis until death from any cause or last follow-up.

### 2.2. Method

Sections of 5 μm thickness were taken from different blocks containing the invasive tumor and non-tumor benign breast parenchyma of the cases and placed on 2–10 lysine slides. The areas that best reflected the invasive tumor and the benign breast parenchyma were marked. They were then removed manually from these slides by using the tip of a number 11 scalpel and placed in a sterile 1.5 mL Eppendorf tube. The tissues were first deparaffinized. Later, RNA extraction was performed by using the ‘*INVITROGEN Recover All Total Nucleic Asid Isolation Kit Thermo Scientific/Ambion, Austin, TX, USA catalog no. AM1975*’. The concentration and quality of the RNAs were measured and recorded with the ‘*Nanodrop 1000, Thermo Scientific, Waltham, MA, USA*’ spectrophotometer device. c-DNA synthesis was performed from the 10-nanogram total RNA of all samples using the Veriti 96-well thermal cycler *(ABI, Applied Biosystems, Foster City, CA, USA)* with 5× primer inside the *TaqmanMicroRna Reverse transcription kit (Thermo Scientific/Ambion, USA, catalog no. 4366596)* and the *TaqMan^®^ Small RNA Assay (Thermo Scientific/Ambion, USA, catalog no. 4427975)*. Synthesized c-DNA was prepared in 96-well plates by using fluorescence-marked 20× primer in the ‘*TaqMan MicroRNA Assay (Thermo Scientific/Ambion, USA, catalog no. 4427975)*’ and ‘*TaqMan Universal PCR Master Miks (Thermo Scientific/Ambion, USA, catalog no. 4304437)*’. The plates were centrifuged for 1 min and loaded into a real-time PCR device for the qRT-PCR reaction. The amplification procedure was performed by adjusting the reaction volume of the 7500 real-time PCR device to 20 μL. Fluorescent radiation amounts were measured in the 7500 real-time PCR device with the 7500 software v2.0.6 for 40 cycles. The CT (cycle threshold) value was determined on the same device for each sample.

The ΔΔCT method was used for qRT-PCR normalization. Rnu6b, which was selected as the reference miRNA, was used for endogenous control. Using the obtained CT values, the ΔΔCT of the samples (ΔΔCT = (CT value of target miRNA of tumor tissue−CT value of endogenous control miRNA (Rnu6b) of tumor tissue)−(CT value of target miRNA of normal tissue−CT value of control miRNA (Rnu6b) of endogenous normal tissue)) value was calculated. The *fold change*, which is the change in the expression level of the target miRNA in tumor tissue compared to normal tissue, was calculated with the formula “2-ΔΔCT”.

### 2.3. Statistical Analysis

The data obtained from the study were analyzed statistically using SPSS version 20.0 software. Descriptive statistics were presented as mean and standard deviation for scalar variables and percentages for categorical ones. Log_2_ transformation was performed for miRNA expression levels. All statistical analysis was run with the transformed values. Differences of variables between the two groups were studied by Student’s t test, whereas one-way ANOVA was used for multiple groups. Cut-off values for miRNA expressed at different levels compared to controls were determined by the receiver operating characteristics (ROCs) curve analysis. The confidence level for statistical significance was defined as 0.95 (α = 0.05).

## 3. Results

We investigated expression level differences in miR-21 and miR-let7f in tumor tissues, and the relationship of these miRNAs with clinicopathological parameters among the three groups in this study. No significant difference was present among the groups in terms of clinicopathological characteristics such as patient age, tumor size, nuclear grade, histological grade, presence/absence of ductal carcinoma in situ, angiolymphatic invasion and perineural invasion status, axillary lymph node metastasis status, microcalcification, Ki-67 proliferation index, disease-free survival time, presence of metastases, and survival status (*p* > 0.05) (Table 1). When the two groups with NEFs were combined, a high miR-21 expression level (2.61 ± 1.62) was found to be associated with progesterone receptor (PR) negativity (*p* = 0.012) (Table 2).

When the groups were compared among themselves in terms of miR-21 expression, the higher expression in Group 3 (mean: 1.84 ± 1.31) was more prominent than in Group 1 (mean: 0.24 ± 1.35), and this difference was statistically significant (*p* < 0.05). When they were compared in terms of miR-let7f expression, the lower miR-let7f expression in Group 2 (−0.98 ± 0.71) was more prominent than in Group 3 (−0.21 ± 1.00), and this difference was statistically significant (*p* < 0.05) (Table 3).

All ofthe cases with NEFs (Group 1 + Group 2) were compared with the IDC cases (Group 3) in terms of miR-21 and let7f expression levels. The mean miR-21 expression level was lower in the BC-NEFs group (0.63 ± 1.19) than the IDC group (1.84 ± 1.31), and the difference was statistically significant (*p* < 0.05). The lower miR-let7f expression level was more prominent in the BC-NEFs group (−0.72 ± 0.82) than in the IDC group (−0.21 ± 1.00), and this difference was again statistically significant (*p*< 0.05) (Table 4).

**Figure 3:**
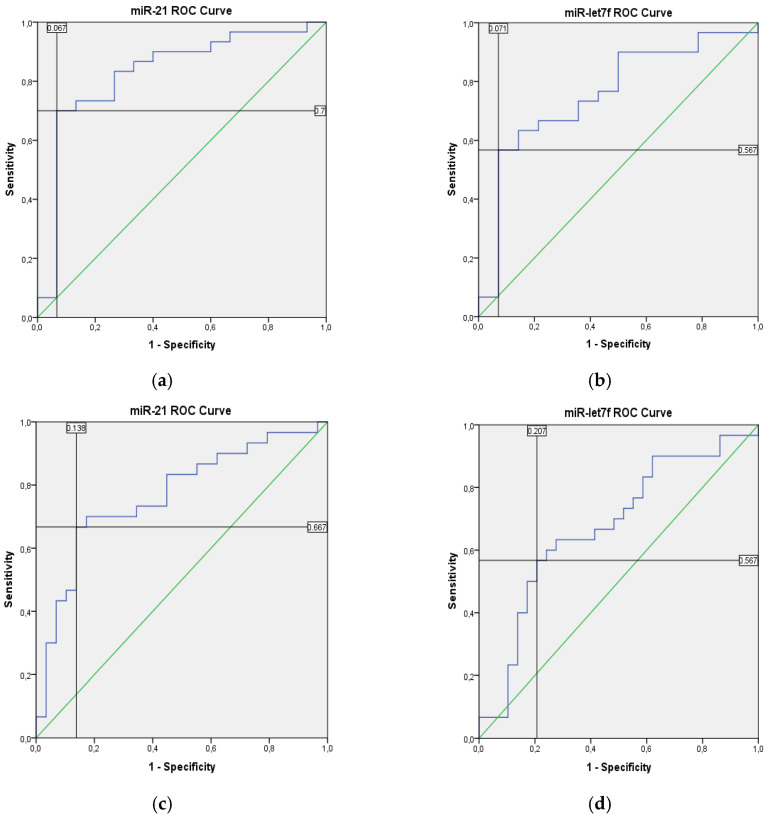
ROC curves. The cut-off values for miR-21 and miR-let7f that can be used to differentiate (**a**) Group 1 (>50% NEFs) from Group 3 (IDC): as regards the identified cut-off values, miRNA-21 was found to have 70% sensitivity and 93.3% specificity between Group 1 and Group 3 cases; (**b**) Group 2 (10–50% NEFs) from Group 3 (IDC): miR-let7f was observed to have 56.7% sensitivity and 92.9% specificity for this identified cut-off value; (**c**,**d**) BC with NEFs from Group 3 (IDC): miR-21 was found to have 66.7% sensitivity and 86.2% specificity between NEBC and IDC cases while miR-let7f was found to have 56.7% sensitivity and 79.3% specificity for identified cut-off values. Blue: ROC curve; Green: Reference line; Black: Reflections of cut-point on x and y lines.

The cut-off value for miRNAs with significant differences in expression levels between the groups was identified with the ROC (receiver operating characteristic) curve analysis (Figure 3).

## 4. Discussion

We investigated the differences in miR-21 and miR-let7f expression levels in tumor tissue between BC-NEFs cases and IDC cases with a similar molecular phenotype and clinical stage, and the relationship with clinicopathological characteristics in this study. miR-21 and miR-let7f are the two miRNAs that have been most studied in human materials and whose oncogenic effect and tumor-suppressor effect are best known. Additionally, miRNA let 7f is a miRNA whose expression level differences have been demonstrated in some solid organ-derived neuroendocrine tumors (except breast cancer) in humans. Therefore, we decided to search these two miRNAs in our study. There are many studies evaluating miRNA expression levels to be used in the diagnosis and clinical follow-up of breast cancer [23,24,25,26,27]. However, as far as we know, there is no study in the literature that reveals the difference between the presence and extent of neuroendocrine differentiation in BC-NEFs and IDC in terms of miRNA expression levels.

miR-21, which is one of the miRNAs we selected for this study, is the most commonly studied miRNA in breast carcinomas and the one with the best known oncogenic role and relationship with an aggressive clinical course [28,29,30]. Although it is effective forthe regulation of many genes, it especially regulates the development of breast carcinoma by targeting the mRNAs of tumor-suppressor genes such as *TPM1*, *PDCD4*, *MARCKS*, *Mapsin*, and *PTEN*. Increased miRNA-21 expression has a negative effect on the expression of *PDCD4*, a tumor-suppressor gene, at the post-transcriptional level, and thus causes metastasis of the breast cancer cell [31]. miR-21 also regulates genes such as *RECK* and *TIMP3*, which are tumor suppressors and matrix metalloproteinase inhibitors, and which also have roles in apoptosis, invasion, and cell migration in breast carcinoma [32].

Lorio et al. have investigated 245 different miRNAs in tumor tissue and normal tissue with the microarray method in breast cancer. An increase in the expression levels of miR-21 and miR-155 in tumor tissue and a decrease in others (miR-10b, miR-125b, and miR-145) were found. Based on these findings, it was reported that miR-21 and miR-155 had an oncogenic effect in breast cancer [33].

Elghoroury et al.haveinvestigated the levels of miR-21 and miR-let7 in the blood in 125 cases, including 50 invasive breast carcinomas, 50 disease-free controls, and 25 benign breast lesions. The level of miR-21 measured in the blood of breast cancer patients was found to be high and the level of miR-let7 to be low, and a low miR-let7 level was found to be associated with the development of metastasis in breast cancer [26]. Anwar et al. have investigated the miR-21 levels in the circulation in 102 breast carcinomas and 15 healthy patients and reported that the miR-21 expression level was increased in breast carcinoma cases and associated with an unfavorable clinical course [27].

We are not aware of any study in the literature investigating the relationship of miR-21 with BC-NEFs. However, studies investigating the relationship between miR-21 and neuroendocrine neoplasms of other organs have been reported [34,35]. An increase in miR-21 expression levels was observed in lung and pancreatic neuroendocrine tumors as well as thyroid medullary carcinoma where miR-21 expression levels in tissue were investigated [36,37]. Lee et al. have investigated the expression profile of miR-21, miR-155, and miR-let7a in 63 lung neuroendocrine neoplasia cases and found that miR-21 and miR-155 showed significantly higher expression in patients with neuroendocrine carcinoma than in patients with neuroendocrine tumors. They also reported a positive correlation between miR-21 expression and lymph node metastasis in cases with neuroendocrine tumors [36]. The miR-21 expression level was higher in tumor tissue compared to normal tissue in three different breast carcinoma subgroups in our study, consistent with the literature. However, the increase in miR-21 level was significantly higher in the IDC group than in BC-NEFs.

The miR-let7 family, which was one of the first miRNAs to be discovered, is a tumor-suppressor miRNA reported to be downregulated in many tumors such as lung, stomach, colon, and breast cancers [38]. The miR-let7 family includes many subgroups such as let-7a, let-7b, let-7c, let-7d, let-7e, let-7f, let-7g, let-7i, miR-98, and miR-202 [39]. RKIP (Raf kinase inhibitory protein) has been reported to inhibit the metastatic invasion of breast cancer cells and repress bone metastasis by inhibiting the MAPK, G protein-associated receptor kinase-2, and NF-kB signaling pathways in various studies investigating the relationship of miR-let7 andbreast carcinoma [40]. MAPK inhibition decreases LIN28 transcription by MYC while the downregulation of LIN28, which is a miR-let-7 biogenesis inhibitor, increases miR-let-7 expression in breast cancer cells. As a result, the expression of HMGA2, which is a chromatin-remodeling protein that activates proinvasive and prometastatic genes such as SNAIL, is decreased. In conclusion, RKIP represses invasion and metastasis through the MAPK, MYC, LIN28, and let-7 cascades and downstream target proteins of let-7 such as HMGA2 [41]. A meta-analysis was conductedfor the potential diagnostic accuracy of the let-7 family in the early diagnosis of several tumors [42]. In this study, it was shown that the let-7 family had high sensitivity and specificity, especially in breast cancer diagnosis.

miR-let7f has been reported as a tumor suppressor whose expression level is decreased in neuroendocrine neoplasms in some other studies [23,24,43]. Dossing et al. have reported a decrease in the expression levels of miR-let7 family members and miR-129-5p in tumor tissues compared to normal tissues in their study involving the tissues of six primary and six metastatic small bowel neuroendocrine neoplasms and four normal small intestines. The inhibition of miR-let7 has been reported to cause bone metastasis by increasing the level of the *BACH1* gene and its targets, MMP1 and HMGA2 [23]. The effect of somatostatin analogs, used in the treatment of neuroendocrine tumors, on miRNAs was investigated in another study by Dossinget al. The study found an increase in their expression in tissue samples, especially for miR-let7 family members, and in miR-148a levels after treatment with somatostatin analogues [24]. Rahmanet al.have investigated the expression levels of HMGA-1 and HMGA-2 in addition to their relationship with miR-let7f in 55 tissue samples of gastroenteropancreatic neuroendocrine neoplasia. Accordingly, an increased expression level of HMGA-1 was observed in 69% of the cases and of HMGA-2 in 73% of the cases. Despite the overexpression of HMGA proteins, miR-let7 was found to be downregulated in 63% of the cases when compared with normal tissue [43]. miR-let7f, which is one of the miR-let7 family members, has been investigated in neuroendocrine neoplasia of organs other than the breast, and there are various findings indicating that it could be associated with neuroendocrine differentiation [23,24,43]. To the best of our knowledge, there areno data in the literature on miR-let7f levels in BC-NEFs. The expression of miR-let7f, which is known to have a tumor-suppressor effect, was decreased in all breast cancer subgroups in our study, in line with the literature. This decrease was significantly more pronounced in BC-NEFs. Considering that tumor tissue contains NEFs in an invasive breast carcinoma, this group of patients may benefit from treatments targeted to increase miR-let7f levels.

Molecular classification of breast carcinomas is performed with immunohistochemical staining, including estrogen, progesterone, Cerbb2, and the Ki-67 index in routine pathology practice. Accordingly, molecular subgroups consist of four main groups knownas “Luminal A, Luminal B, Human epidermal growth factor receptor 2 (HER2) overexpressing, and the triple negative type” [44]. Traditional prognostic factors in breast cancer are not successful in predicting the prognosis in every patient due to individual differences and tumor heterogeneity [45]. When we combined two groups of tumors with NEFsin this study, we found that a high miR-21 expression level was associated with progesterone receptor negativity. There are several studies in the literature where the relationship between miR-21 expression levels and clinicopathological parameters in breast carcinoma were investigated [27,46,47]. Lee et al. have investigated the miR-21 expression level in tumor and normal tissue in 109 breast cancer cases. Accordingly, as the miR-21 expression level increases, the tumor diameter, histological grade, stage, and Ki-67 proliferation index also increase. Additionally, this expression increase was found to be associated with ER negativity, HER-2 positivity, and a higher risk of death [46]. Yadav et al. have investigated the miR-21 level in circulation in 75 patients with breast carcinoma. Accordingly, an increase in the miR-21 expression level was found to be associated with an increase in the risk of tumor stage, lymph node metastasis, and distant metastasis [47]. Contrary to this, Anwar et al. investigated the miR-21 level in circulation in 102 breast cancer patients and 15 healthy women and found no significant difference between the miR-21 expression level and tumor stage, tumor diameter, histological grade, and molecular subtype [27]. Consecutive IDC cases with molecular phenotype and stage similar to the cases with BC-NEFs were identified while forming the groups in our study. Thus, an attempt was made to minimize the miRNA expression level differences that may arise from tumor stage or the different molecular subtypes. However, there were some limitations of the current study such as retrospective design, small number of cases, lack of functional experiments to confirm the biological effects of miR-21 and miR-let7f in BC-NEFs, and no validation with external datasets (such as GEO). Other miRNAs or molecular pathways that may be relevant to BC-NEFs pathogenesis could be investigated in future studies.

In conclusion, our findings suggest that a decrease in miR-let7f expression (with tumor-suppressor effects) is prominent in the presence of NEFs in breast tumors, and an increase in miR-21 expression (with oncogenic effects) is more prominent in IDC without neuroendocrine features. This study may encourage the investigation of the expression levels of other neuroendocrine-associated miRNAs in BC-NEFs, most of which display a luminal molecular phenotype.

## Figures and Tables

**Table 1:** The distribution of the clinical and histopathological characteristics of the groups (mean and standard deviation values (O ± SD), range of values (min–max) and distribution percentages).

	Group 1 (*n* = 15)	Group 2 (*n* = 14)	Group 3 (*n* = 30)
Age	O: 69.67 ± 11.78	O: 58.71 ± 12.73	O: 54.20 ± 15.73
	(47–87)	(40–79)	(23–83)
Tumor Size (cm)	Mean: 2.13 ± 0.95	Mean: 2.56 ± 0.74	Mean: 2.44 ± 0.85
	(0.5–4.5)	(1.5–4.00)	(1.2–5.00)
Ki-67 Percentage (%)	Mean: 14.87 ± 8.63	Mean: 34.36 ± 16.82	Mean: 24.54 ± 13.17
	(2–30)	(11–70)	(10–60)
Lymph Node Metastasis	Absent: 60% (*n* = 9)	Absent: 28.6% (*n* = 4)	Absent: 50% (*n* = 15)
	Present: 40% (*n* = 6)	Present: 71.4% (*n* = 10)	Present: 50% (*n* = 15)
Nuclear Grade	1: 6.7% (*n* = 1)	1: 0%	1: 0%
	2: 86.7% (*n* = 13)	2: 42.9% (*n* = 6)	2: 70% (*n* = 21)
	3: 6.7% (*n* = 1)	3: 57.1% (*n* = 8)	3: 30% (*n* = 9)
Histological Grade	1: 6.7% (*n* = 1)	1: 0%	1: 3.3% (*n* = 1)
	2: 93.3% (*n* = 14)	2: 57.1% (*n* = 8)	2: 70% (*n* = 21)
	3: 0%	3: 42.9% (*n* = 6)	3: 26.7% (*n* = 8)
Angiolymphatic Invasion	Absent: 60% (*n* = 9)	Absent: 21.4% (*n* = 3)	Absent: 46.7% (*n* = 14)
	Present: 40% (*n* = 6)	Present: 78.6% (*n* = 11)	Present: 53.3% (*n* = 16)
Perineural Invasion	Absent: 100% (*n* = 15)	Absent: 78.6% (*n* = 11)	Absent: 83.3% (*n* = 25)
	Present: 0%	Present: 21.4% (*n* = 3)	Present: 16.7% (*n* = 5)
In Situ Component	Absent: 20% (*n* = 3)	Absent: 7.1% (*n* = 1)	Absent: 20% (*n* = 6)
	Present: 80% (*n* = 12)	Present: 92.9% (*n* = 13)	Present: 80% (*n* = 24)
Microcalcification	Absent: 53.3% (*n* = 8)	Absent: 50% (*n* = 7)	Absent: 50% (*n* = 15)
	Present: 46.7% (*n* = 7)	Present: 50% (*n* = 7)	Present: 50% (*n* = 15)
Molecular Subtype	Lum A: 66.7% (*n* = 10)	Lum A: 21.4% (*n* = 3)	Lum A: 46.7% (*n* = 14)
	Lum B: 33.3% (*n* = 5)	Lum B: 78.6% (*n* = 11)	Lum B: 53.3% (*n* = 16)
Clinical Stage	Early: 73.3% (*n* = 11	Early: 42.9% (*n* = 6)	Early: 56.7% (*n* = 17)
	Late: 26.7% (*n* = 4)	Late: 57.1% (*n* = 8)	Late: 43.3% (*n* = 13)
Disease-Free Survival (months)	Mean: 113.66 ± 9.35	Mean: 120.97 ± 9.29	Mean: 122.83 ± 9.01
	min-max: 7–128	min-max: 22–135	min-max: 13–152
Overall Survival (months)	Mean: 88.34 ± 12.46	Mean: 123.29 ± 811	Mean: 129.83 ± 7.5
	Median: 71	Median: 104	Median: 133
	(min-max: 7–133)	(min-max: 25–135)	(min-max: 13–152)
Metastasis	Absent: 86.7% (*n* = 13)	Absent: 85.7% (*n* = 12)	Absent: 73.3% (*n* = 22)
	Present: 13.3% (*n* = 2)	Present: 14.3% (*n* = 2)	Present: 26.7% (*n* = 8)
Metastasis site	Case 1: bone, liver, lung	Case 1: bone, liver	Case 1: bone, brain
			Case 2: bone
			Case 3: bone, brain
			Case 4: bone, liver
	Case 2: bone, liver	Case 2: bone	Case 5: bone, lung
			Case 6: bone
			Case 7: bone
			Case 8: bone, lung
Exitus (survival status)	Absent: 53.3% (*n* = 8)	Absent: 85.7% (*n* = 12)	Absent: 70% (*n* = 21)
	Present: 46.7% (*n* = 7)	Present: 14.3% (*n* = 2)	Present: 30% (*n* = 9)

**Table 2:** Correlation between miR-21 and miR-let7f expressions and clinicopathologic characteristics in 29 cases with BC-NEFs.

Clinicopathologic Characteristics	No. of Patients (*n* = 29)	miR-21 *	miR-let7f *
Age			
<45	3	1.20 ± 1.43	−0.23 ± 0.93
>45	26	0.56 ± 1.18	−0.78 ± 0.81
*p*		0.387	0.275
Tumor Size (cm)			
<2 cm	13	0.47 ± 1.43	−0.61 ± 0.99
2 cm< and >5 cm	16	0.76 ± 1	−0.81 ± 0.67
*p*		0.521	0.506
Ki-67 Percentage (%)			
<20%	13	0.68 ± 1.19	−0.55 ± 0.91
>20%	16	0.58 ± 1.23	−0.86 ± 0.74
*p*		0.828	0.320
Lymph Node Metastasis			
Absent	13	0.64 ± 1.43	−0.80 ± 0.80
Present	16	0.62 ± 1.01	−0.66 ± 0.85
*p*		0.965	0.647
Nuclear Grade			
1	1	−0.63	−1.28
2	19	0.41 ± 1.28	−0.66 ± 0.88
3	9	1.22 ± 0.77	−0.79 ± 0.74
*p*		0.137	0.745
Histological Grade			
1	1	−0.62	−1.27
2	22	0.62 ± 1.20	−0.61 ± 0.90
3	6	0.83 ± 1.24	−1.01 ± 0.34
*p*		0.544	0.461
Angiolymphatic Invasion			
Absent	12	0.71 ± 1.28	−0.53 ± 0.99
Present	17	0.57 ± 1.16	−0.86 ± 0.67
*p*		0.757	0.289
Perineural Invasion			
Absent	26	0.53 ± 1.19	−0.68 ± 0.84
Present	3	1.47 ± 1.01	−1.07 ± 0.60
*p*		0.202	0.448
ER			
Negative	0		
Positive	29	0.63 ± 1.19	−0.72 ± 0.82
*p*		n/a	n/a
PR			
Negative	2	2.61 ± 1.62	−0.03 ± 1.70
Positive	27	0.48 ± 1.05	−0.77 ± 0.76
*p*		0.012	0.225
HER			
Negative	25	0.46 ± 1.19	−063 ± 0.84
Positive	4	1.64 ± 0.65	−1.27 ± 0.40
*p*		0.066	0.153
Metastasis			
Absent	25	2.46 ± 2.58	0.75 ± 0.53
Present	4	0.54 ± 0.25	0.55 ± 0.28
*p*		0.154	0.476

* Normalized Ct value of miRNA (mean ± standard deviation). n/a: not applicable; *p*: *p* value.

**Table 3:** The comparison of the groups in terms of miR-21 and miR-let7f expression levels.

	Group 1 * (*n* = 15)	Group 2 * (*n* = 14)	Group 3 * (*n* = 30)	*p*
miR-21	O:0.24 ± 1.35	O:1.04 ± 0.86	O:1.84 ± 1.31	*p* = 0.001
	(−2.24–3.76)	(−0.77–2.58)	(−0.98–5.51)	Group 3 > Group 1
miR-let7f	O: −0.48 ± 0.86	O: −0.98 ± 0.71	O: −0.21 ± 1.00	*p* = 0.03
	(−1.91–1.17)	(−1.85–0.84)	(−1.81–2.51)	Group 2 > Group 3

* Group 1: BC cases with NE features in >50% of the tumor. * Group 2: BC cases with NE features in 10–50 % of the tumor. * Group 3: No NE features in tumor.

**Table 4:** The comparison of the BC-NEFs (Group 1 + Group 2) and IDC groups in terms of miR-21 and miR-let7f expression levels.

	BC-NEFs (*n* = 29)	IDC (*n* = 30)	*p*
miR-21	O: 0.63 ± 1.19	O: 1.84 ± 1.31	*p* = 0.0005
	(−2.24–3.76)	(−0.98–5.51)	
miR-let7f	O: −0.72 ± 0.82	O: −0.21 ± 1.00	*p* = 0.04
	(−1.91–1.17)	(−1.81–2.51)	

## Data Availability

The original contributions presented in the study are included in the article, further inquiries can be directed to the corresponding author.
